# Commentary: What Is Art Good For? The Socio-Epistemic Value of Art

**DOI:** 10.3389/fnhum.2017.00602

**Published:** 2017-12-08

**Authors:** Martin Skov, Marcos Nadal

**Affiliations:** ^1^Danish Research Centre for Magnetic Resonance, Copenhagen University Hospital, Copenhagen, Denmark; ^2^Center for Decision Neuroscience, Copenhagen Business School, Frederiksberg, Denmark; ^3^Human Evolution and Cognition Group (EvoCog), University of the Balearic Islands and IFISC, Palma de Mallorca, Spain

**Keywords:** neuroaesthetics, empirical aesthetics, art, cognitive neuroscience, social neuroscience

*Art, in fact, can be nothing but violence, cruelty, and injustice*.

—Marinetti, *I manifesti del Futurismo*

Does neuroaesthetics have a problem? Sherman and Morrissey (2017) criticize the field for focusing narrowly on how art elicits pleasurable responses, and for neglecting its social relevance and impact. Neuroaesthetics, they argue, reduces the experience of art to isolated individuals' ratings in artificial lab settings, and ignores “socially-relevant outcomes of art appreciation or the social context of art creation and art appreciation.” Consequently, it fails to “capture or appreciate the social, cultural, or historical situatedness of the art-object or the person whose experience is being studied.”

There is no question that we know little about the social aspect of art behavior and its underlying psychological and neurobiological mechanisms. Because art is often a transient phenomenon created as function *of* a social act, as in music, dance, or performance, the features of collective settings surely modulate cognition and affect. Dance, for instance, can coordinate emotional responses to promote social cohesion (Vicary et al., 2017). Nevertheless, the precise way in which social settings influence brain activity when experiencing art remains largely unknown.

We know of no neuroaestetician who would not welcome research on the psychology and biology of art behavior in social contexts. Yet, Sherman and Morrissey (2017) portray neuroaesthetics as dismissing such research topics and promoting an a-social conception of art experience. They fault neuroaesthetics for “conflating the art with aesthetics,” for having “privileged investigating individual judgments of beauty or preference,” for construing art appreciation as a “passive reception of perceptual information from art-objects,” and for discounting “what many would consider the very essence of art: its communicative nature, its capacity to encourage personal growth (…), to challenge preconceptions (…), and to provide clarity on ambiguous concepts or ideas.”

This is a misrepresentation of neuroaesthetics. We have refuted these and other similar contentions extensively elsewhere (Pearce et al., 2016). Here we only have space to make three points. First, although many neuroaesthetics studies do aim to understand how the brain constructs aesthetic value, it is hardly the only aspect of art experience being investigated. Much psychological and neuroscientific research on art actually concerns perception and representation, not valuation (e.g., Bromberger et al., 2011; Pegors et al., 2015; Choo et al., 2017). Second, neuroaesthetics does not discount the social dimension of art experience. Music research, for instance, has uncovered that people compute tones using expectations set by music's tonal system and internalized as members of a social community (Brattico and Pearce, 2013; Egermann et al., 2013). Third, contrary to Sherman and Morrissey's (2017) claims, researchers have adapted their experimental paradigms to social art contexts, including museums, concert halls, and theaters (e.g., Tschacher et al., 2012; Egermann et al., 2013; Jola and Grosbras, 2013; Brieber et al., 2014; Vicary et al., 2017).

These and other ongoing studies prove that the factor limiting scientific research of art in its social milieu is not one of principle, but one of technological availability. Technology (e.g., eye trackers, physiological monitoring) is becoming cheaper and increasingly portable, enabling the study of art in its social context. Behind this misleading picture of neuroaesthetics we detect another, perhaps the true, goal of Sherman and Morrisey's paper: to defend a specific conception of what art is—or should be. At the heart of their criticism lurks an assumption: that art is a force for good—for social good, moral good. Sherman and Morrisey presume that art can make us better people: “engaging with art can be potentially transformative, for it encourages us to consider the welfare and good of other people.”

It is this assumption that explains the peculiar choice of purported social functions they urge neuroaesthetics to study: to enhance self-understanding and to enhance the understanding of others. But Sherman and Morrissey's (2017) claim rests only on their own intuition, not on empirical evidence showing that art actually does improve us as moral beings. They are upfront about this fact, basing their case for both proposed functions of art solely on philosophical authority. An empirical science of art, however, cannot be motivated solely by armchair assumptions about the nature of art. Philosophy and art history abound with views contrary to the notion that art is a force for social good. Plato vilified art for being unable to produce true ideas, and for emotionally enticing and contaminating the innocent minds of Athens's youth. Innumerable thinkers, parents, and rulers, in the wake of Plato, have viewed art more as a force of social evil than of social good. Can we base a proper empirical science of human art experience on whatever intuitions and assumptions we find most appealing?

Art has come to occupy a problematic place in psychology and neuroscience. Several recent papers have seen fit to propose behavioral, cognitive and emotional traits/processes as integral to art experience. However, such proposals are largely unconstrained by knowledge about cognition and neuroscience, and rarely backed up by evidence (e.g., Bullot and Reber, 2013; Christensen, 2017; Menninghaus et al., 2017; Pelowski et al., 2017). As an object of research, art is so broad and imbued with centuries of philosophical speculation that any claim seems to have an aura of validity. Art appears to be good-for everything: it can be imputed with whatever feature or function strikes a researcher's fancy. Whether such feature or function is supported by empirical evidence, or is in accordance with current knowledge about human brain and cognition, seems only a secondary concern. To advance as a scientific discipline, neuroasthetics must do better.

## Author contributions

All authors listed have made a substantial, direct, and intellectual contribution to the work, and approved it for publication.

### Conflict of interest statement

The authors declare that the research was conducted in the absence of any commercial or financial relationships that could be construed as a potential conflict of interest.
